# High willingness to use rapid fentanyl test strips among young adults who use drugs

**DOI:** 10.1186/s12954-018-0213-2

**Published:** 2018-02-08

**Authors:** Maxwell S. Krieger, Jesse L. Yedinak, Jane A. Buxton, Mark Lysyshyn, Edward Bernstein, Josiah D. Rich, Traci C. Green, Scott E. Hadland, Brandon D. L. Marshall

**Affiliations:** 1 Department of Epidemiology, Brown University School of Public Health, 121 South Main Street, Box G-S-121-2, Providence, RI 02912 USA; 2 School of Population and Public Health, University of British Columbia, Vancouver, British Columbia Canada; 3 British Columbia Centre for Disease Control, Vancouver, British Columbia Canada; 4 Vancouver Coastal Health, Vancouver, British Columbia Canada; 5 Department of Emergency Medicine, Grayken Center for Addiction, Boston University School of Medicine, Boston, MA USA; 6 Department of Pediatrics, Grayken Center for Addiction, Boston Medical Center, Boston, MA USA; 7 Department of Emergency Medicine, Warren Alpert Medical School of Brown University, Providence, RI USA

**Keywords:** Overdose, Opioids, Fentanyl, Harm reduction, Willingness

## Abstract

**Background:**

Synthetic opioid overdose mortality among young adults has risen more than 300% in the USA since 2013, primarily due to the contamination of heroin and other drugs with illicitly manufactured fentanyl. Rapid test strips, which can be used to detect the presence of fentanyl in drug samples (before use) or urine (after use), may help inform people about their exposure risk. The purpose of this study was to determine whether young adults who use drugs were willing to use rapid test strips as a harm reduction intervention to prevent overdose. We hypothesized that those who had ever overdosed would be more willing to use the test strips.

**Methods:**

We recruited a convenience sample of young adults who use drugs in Rhode Island from May to September 2017. Eligible participants (aged 18 to 35 with past 30-day drug use) completed an interviewer-administered survey. The survey assessed participant’s socio-demographic and behavioral characteristics, overdose risk, as well as suspected fentanyl exposure, and willingness to use take-home rapid test strips to detect fentanyl contamination in drugs or urine. Participants were then trained to use the test strips and were given ten to take home.

**Results:**

Among 93 eligible participants, the mean age was 27 years (SD = 4.8), 56% (*n* = 52) of participants were male, and 56% (*n* = 52) were white. Over one third (*n* = 34, 37%) had a prior overdose. The vast majority (*n* = 86, 92%) of participants wanted to know if there was fentanyl in their drug supply prior to their use. Sixty-five (70%) participants reported concern that their drugs were contaminated with fentanyl. After the brief training, nearly all participants (*n* = 88, 95%) reported that they planned to use the test strips.

**Conclusions:**

More than 90% of participants reported willingness to use rapid test strips regardless of having ever overdosed, suggesting that rapid fentanyl testing is an acceptable harm reduction intervention among young people who use drugs in Rhode Island. Study follow-up is ongoing to determine whether, how, and under what circumstances participants used the rapid test strips and if a positive result contributed to changes in overdose risk behavior.

## Background

North America is in the midst of an unprecedented overdose epidemic. In 2016, provisional data indicates that overdose mortality increased over 20% from 2015 to reach a record high of 64,070 deaths [1, 2]. The opioid overdose epidemic, once dominated by prescription opioid misuse, is now driven by the use of heroin and other illicit drugs [3–6]. Heroin-related deaths have quadrupled since 2010, while prescription opioid deaths have increased only slightly [7, 8]. Although diversion of pharmaceutical fentanyl, such as transdermal patches, is an ongoing problem, in recent years, illicitly manufactured fentanyl and related compounds have been regularly mixed into heroin, cocaine, and counterfeit prescription pills [9–13]. Since the widespread introduction of illicitly manufactured fentanyl into the drug supply in North America in 2013 [11], fentanyl-related deaths have sharply increased [8, 14]. In the United States (US), six states that publish data on fentanyl-related fatalities reported that the number of overdose deaths attributable to fentanyl increased by over 350% between 2013 and 2014, from 392 to over 1400 [15]. In British Columbia, Canada, fentanyl-related overdose deaths increased over 600% from 2014 to 2016, with fentanyl being detected in 67% of all overdose-related deaths in 2016 compared to 25% in 2014 [16].

Young adults have experienced the greatest increases in fentanyl overdose mortality as heroin and counterfeit pill use has surged over the last decade [17, 18]. In Rhode Island, a state with the fifth highest overdose rate in the US, over half of all fentanyl overdose deaths in 2014 and 2015 were among individuals under the age of 35 [19]. Drug-using young adults may have less experience with overdose prevention strategies and may engage in drug-using behaviors that put them at higher risk for overdose, such as polysubstance use or combined alcohol and drug use [14]. Moreover, harm reduction services such as needle distribution programs are currently underutilized by the majority of young adults who use drugs, due in part to the stigma associated with using such services [20, 21].

People who use drugs may not know if fentanyl is being cut into their drugs and might rely on ineffectual information regarding smell, taste, color, and word of mouth to determine the presence of fentanyl [22]. To address the alarming public health challenges associated with illicitly manufactured fentanyl, recent innovations in rapid drug testing technology may promote risk reduction behaviors among young adults who are at high risk of fentanyl overdose. Rapid fentanyl test strips, such as those recently piloted at a supervised injection facility (SIF) in Vancouver, Canada, are used to detect the presence of fentanyl in drug samples or urine [23, 24]. A pilot study at the Vancouver SIF found a high positivity rate of fentanyl-contaminated drugs and that clients who used the strips prior to consumption reduced their dose and decreased the risk of overdose [24]. In this study, we sought to determine whether young people (aged 18 to 35) who use drugs in Rhode Island were willing to use take-home rapid fentanyl test strips as a harm reduction intervention to prevent accidental overdose due to fentanyl contamination. We hypothesized that young adults would report high interest in and willingness to use rapid fentanyl testing and that those who had ever overdosed would be more willing to use rapid fentanyl test strips than young adults with no prior overdose experience.

## Methods

### Study design

**Fig. 1:**
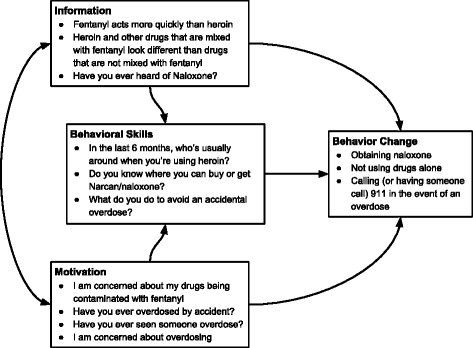
Selected information, behavior skills, and motivation questions with behavioral change outcomes

This pilot study was conducted to determine the feasibility, acceptability, and behavioral outcomes associated with take-home rapid fentanyl tests as a harm reduction intervention to prevent accidental overdose among drug-using young people. Our study was guided by the information-motivation-behavioral (IMB) skills model of engagement in health behaviors [25]. The IMB model hypothesizes that if a person possesses the information, motivation, and behavioral skills to act, there is an increased likelihood that she/he will fulfill and maintain the desired behaviors [25, 26]. The combination of increased information, motivation, and behavioral skills is necessary to produce desired behavior change (in this case, overdose risk reduction practices). We hypothesized that having experienced an overdose would lead to increased behavioral skills to avoid an overdose (see Fig. 1) and would thus be associated with an increased willingness to use fentanyl testing strips. The IMB model informed the questions included on the interviewer-administered survey, which assessed participant knowledge about fentanyl and overdose prevention (information), desire to increase their knowledge of fentanyl or concern about overdose (motivation), and overdose prevention skills based on experience with overdose (behavioral skills) [25]. We also included questions assessing socio-demographic characteristics, overdose risk, fentanyl exposure, and willingness to use take-home rapid test strips to detect fentanyl contamination in participant’s drug supply or urine.

Survey questions were further informed by our previous 2015 study, the Rhode Island Young Adult Prescription and Illicit Drug Study (RAPIDS), which demonstrated that non-pharmaceutical fentanyl exposure was a significant problem for young people who use drugs in Rhode Island [27].

### Recruitment and eligibility criteria

Beginning in May 2017, we recruited participants from the 2015 RAPIDS study, 61% of whom previously agreed to be contacted for future research studies. The eligibility criteria for RAPIDS included being aged 18 to 29, residing in Rhode Island, not currently being in alcohol or substance abuse treatment programs, and reporting past 30-day non-medical use of prescription opioids. From May to September 2017, we recruited additional young adults who use drugs in Rhode Island through internet advertisements (e.g., Craigslist and Reddit), digital bus advertisements, public canvassing, and word of mouth. Eligibility criteria for the current study included (1) living in Rhode Island at the time of enrollment, (2) aged 18 to 35 at the time of enrollment, and (3) self-reported past 30-day heroin or cocaine use, injection drug use, or having purchased prescription pills on the street. We expanded the age and drug use eligibility criteria created based on our recent research of the fentanyl overdose epidemic in Rhode Island [19].

### Survey and fentanyl test strip protocols

Surveys were administered by a professionally trained research assistant using a Qualtrics web-based survey tool after obtaining written consent. The surveys took an average of 1 h to complete. Research assistants were trained in harm reduction techniques such as recognizing and responding to overdose and administering naloxone. Research assistants were also trained on how to use and interpret rapid fentanyl test strips. Based on prior research at an SIF in Vancouver, we purchased rapid fentanyl urine test strips, which have a detection level of 20 ng/ml [28]. The rapid fentanyl test strips are single-use immunoassay tests for the qualitative detection of fentanyl and norfentanyl [29]. The tests are advertised as being able to detect fentanyl analogs such carfentanil, acetyl fentanyl, and butyryl fentanyl, but further research is needed to determine if the tests are able to detect other novel fentanyl analogs [30]. We purchased the strips in bulk for approximately $1 USD for a single, individually packaged fentanyl test strip.

Once the primary survey was complete, participants then received a brief in-person one-on-one training on how to use rapid fentanyl test strips. During the first part of the training, participants viewed a short instructional video of heroin being tested for fentanyl and another video on how to interpret test strip results. The next part of the training included a plain language handout on how to test urine, powdered drugs, and pills using the test strips. In order to determine the feasibility of both urine and drug sample testing, the first 40 participants were instructed to test their urine (after drug use) to detect the presence of fentanyl, while the remaining participants were instructed to test a sample of their drugs or residue (before consumption). Participants were instructed that a negative result could still mean their drugs contained other fentanyl analogs or drug contaminants and that the tests did not reveal the quantity of fentanyl, only its presence or absence. Participants were asked if they had any questions regarding the videos or handout. Participants were then asked several brief questions about the training, including “I feel confident in my ability to test my own drugs/urine for fentanyl,” and “I feel confident in my ability to read the results of the fentanyl testing strips.” The primary outcome (willingness to use the fentanyl test strips) was assessed with the question, “I plan to use the testing strips,” with response options offered on a 4-point Likert scale of *strongly agree* to *strongly disagree*. Participants were then provided with ten strips to take home and a printout of what a positive or negative result looked like. Participants also received resources which included fentanyl harm reduction suggestions, instructions on how to recognize an opioid overdose, and information on local resources. At the end of the study visit, participants were compensated $25 USD for their time.

We report here the results of the baseline survey and the outcomes of the fentanyl strip test training and receipt of the take-home tests. Data from the participants’ follow-up visits, which surveyed whether participants used the fentanyl rapid test strips and if the intervention resulted in overdose risk behavior change (as hypothesized by the IMB conceptual model), is being analyzed and will be discussed in a subsequent study.

### Statistical analyses

First, response options to the primary study outcomes (e.g., willingness to use the fentanyl testing strips) were re-coded into three categories: “agree” (for answers *strongly agree* or *agree*), “disagree” (for answers *strongly disagree* or *disagree*), and “do not know/refuse” (for answers *do not know* or *refuse*). We then stratified the participants based on the responses to the question, “Have you ever overdosed?” to determine whether people who had ever overdosed were more or less likely to be willing to use take-home fentanyl test strips compared to those who had never overdosed. Respondents who answered “yes” to a lifetime history of overdose were compared to those who answered “no” or “do not know.” We used Pearson’s chi-square test to compare categorical variables and Fisher’s exact test when a cell count was ≤ 5. Two-sided *p* values were used for all variables and were considered statistically significant at 0.05.

## Results

Socio-demographic and substance use characteristics of study participants are summarized in Table 1. A total of four participants were recruited from RAPIDS. Among 93 participants who completed the survey, the mean age was 27 years (SD = 4.8). More than half (56%) of the study participants were male and 56% were white, followed by mixed race (30%) and black (14%). Over half reported that they had ever been homeless (59%), and almost three quarters (73%) reported a lifetime history of arrest. Over half of the participants reported regular non-medical use (defined as at least once a week or every day) of prescription opioids (54%), while approximately one third reported heroin (37%) and cocaine use (37%). Almost half had injected drugs (ever in lifetime 48%, in last 6 months: 42%). Of the 93 participants in this study, 34 (37%) reported that they had ever overdosed. Among the participants who had ever overdosed, 18 (53%) reported ever overdosing on a drug which they thought might have been contaminated with fentanyl. As shown in Table 1, socio-demographic and drug use factors that were significantly associated with having ever overdosed included having ever been arrested, having regular non-medical prescription opioid use, having regular heroin use, having ever injected, having been in an alcohol and drug treatment program, and having ever seen someone overdose (all *p* < 0.01).

**Table 1:** Selected socio-demographic and substance use characteristics of participants who have overdosed

Characteristic	Overall *n* (%) *n* = 93	Never overdosed *n* (%) *n* = 59	Have overdosed *n* (%) *n* = 34	*p* value
Gender				
Male	52 (56%)	33 (55%)	19 (56%)	0.842
Female	37 (40%)	24 (41%)	13 (38%)	
Something else	4 (4%)	2 (4%)	2 (6%)	
Race				
Black	13 (14%)	10 (17%)	3 (9%)	0.369
White	52 (56%)	30 (51%)	22 (65%)	
Mixed or other	28 (30%)	19 (33%)	9 (26%)	
Homeless ever				
Yes	55 (59%)	30 (51%)	25 (74%)	0.034
No	38 (41%)	29 (49%)	9 (26%)	
Homeless last 6 months				
Yes	17 (18%)	12 (20%)	5 (15%)	0.521
No	76 (82%)	47 (80%)	29 (85%)	
Ever arrested				
Yes	68 (73%)	37 (63%)	31 (91%)	0.004
No	25 (27%)	22 (37%)	3 (9%)	
Arrested last 6 months				
Yes	16 (17%)	6 (10%)	10 (29%)	0.025
No	77 (83%)	53 (90%)	24 (71%)	
Ever incarcerated				
Yes	36 (39%)	19 (32%)	17 (50%)	0.098
No	57 (61%)	40 (68%)	17 (50%)	
Ever purchased fentanyl online or the “dark web”				
Yes	5 (5%)	2 (3%)	3 (9%)	0.510
No	88 (95%)	57 (97%)	31 (91%)	
Regular Heroin Use ^a^				
Yes	34 (37%)	16 (27%)	18 (53%)	0.015
No	59 (63%)	43 (73%)	16 (47%)	
Regular cocaine use^a^				
Yes	34 (37%)	19 (32%)	15 (44%)	0.263
No	59 (63%)	40 (68%)	19 (56%)	
Regular non-medical prescription opioid use^a,b^				
Yes	50 (54%)	26 (44%)	24 (71%)	0.015
No	43 (46%)	33 (56%)	10 (29%)	
Ever been prescribed fentanyl				
Yes	4 (4%)	2 (3%)	2 (6%)	0.931
No	89 (96%)	57 (97%)	32 (94%)	
Ever been in alcohol or drug treatment program				
Yes	62 (67%)	33 (56%)	29 (85%)	0.004
No	31 (33%)	26 (44%)	5 (14%)	
Ever injected				
Yes	45 (48%)	20 (34%)	25 (74%)	< 0.001
No	48 (52%)	39 (66%)	9 (26%)	
Injected last 6 months				
Yes	39 (42%)	15 (25%)	24 (71%)	< 0.001
No	54 (58%)	44 (75%)	10 (29%)	
Ever seen someone overdose				
Yes	59 (63%)	29 (49%)	30 (88%)	< 0.001
No	34 (37%)	30 (51%)	4 (12%)	

^a^At least once a week or every day

^b^Includes Percocet, Vicodin, tramadol, OxyContin, oxycodone, hydromorphone, hydrocodone, oxymorphone, or morphine

Information, motivation, and behavioral skills-related factors, stratified by history of overdose, are summarized in Table 2. Almost half of all study participants reported a concern about overdosing (48%), with those who had ever overdosed being significantly more likely to report a concern about overdosing (74%, *p* < 0.01). Nearly three quarters of all participants expressed concern about their drugs being contaminated with fentanyl (70%); however, expressing concern was not significantly associated with participants having ever overdosed. Almost half of the overall sample (47%) was confident that (at some time in the past) they used fentanyl-laced drugs. A greater number of individuals who had ever overdosed (68% vs. 35%, *p* = 0.01) reported confidence that they had used fentanyl-laced drugs. When asked “Do you know where you can buy or get Narcan/naloxone?”, more participants who had ever overdosed reported “yes” (76% vs. 46%, *p* = 0.01). As shown in Table 2, nearly all individuals reported that they wanted to know if fentanyl was in their drugs before taking them (93%), which was not significantly associated with overdose history.

**Table 2:** Selected information-motivation-behavioral (IMB) skills model-related factors associated with overdose among young adults

Characteristic	Overall *n* (%) *n* = 93	Never overdosed *n* (%) *n* = 59	Have overdosed *n* (%) *n* = 34	*p* value
Information				
Fentanyl acts more quickly than heroin				
Agree	50 (54%)	29 (49%)	21 (62%)	0.042
Disagree	10 (11%)	4 (7%)	6 (18%)	
Neutral/do not know	33 (35%)	26 (44%)	7 (20%)	
I would like to be able to know if there is fentanyl in my drugs before I take them				
Agree	86 (93%)	56 (95%)	30 (88%)	0.070
Disagree	5 (5%)	1 (2%)	4 (12%)	
Neutral/do not know	2 (2%)	2 (3%)	0 (0%)	
Heroin and other drugs that are mixed with fentanyl look different than drugs that are not mixed with fentanyl				
True	25 (27%)	16 (27%)	9 (26%)	0.089
False	40 (43%)	21 (36%)	19 (56%)	
Do not know	28 (30%)	22 (37%)	6 (18%)	
Have you ever heard of naloxone?				
Yes	82 (88%)	50 (85%)	32 (94%)	0.082
No	10 (11%)	9 (15%)	1 (3%)	
Do not know	1 (1%)	0 (0%)	1 (3%)	
Motivation				
Concerned about overdosing				
Agree	45 (48%)	20 (34%)	25 (74%)	< 0.001
Disagree	42 (45%)	35 (59%)	7 (21%)	
Neutral/do not know	6 (6%)	4 (7%)	2 (6%)	
Concerned about drugs being contaminated with fentanyl				
Agree	65 (70%)	41 (70%)	24 (70%)	0.686
Disagree	17 (18%)	12 (20%)	5 (15%)	
Neutral/do not know	11 (12%)	6 (10%)	5 (15%)	
Confident have ever used a drug that was laced with fentanyl				
Agree	44 (47%)	21 (35%)	23 (68%)	0.010
Disagree	43 (46%)	34 (58%)	9 (26%)	
Neutral/do not know	6 (7%)	4 (7%)	2 (6%)	
Behavioral Skills				
In the last 6 months, who is usually around when you are using heroin?^a,b^				
I use heroin alone	32 (74%)	15 (79%)	17 (71%)	0.806
A close friend	18 (42%)	7 (37%)	11 (46%)	0.573
A casual friend or acquaintance	16 (37%)	7 (37%)	9 (38%)	0.969
A sex partner	15 (35%)	6 (32%)	9 (38%)	0.704
Other	13 (30%)	5 (26%)	8 (33%)	0.641
What do you do to avoid an accidental overdose?^a^				
Take smaller amounts	56 (60%)	34 (58%)	22 (65%)	0.514
Go slow	55 (59%)	31 (52%)	24 (71%)	0.094
Avoid mixing with other drugs	46 (49%)	34 (58%)	12 (35%)	0.042
Using with someone else	46 (49%)	26 (44%)	20 (59%)	0.180
Avoid mixing with alcohol	39 (42%)	26 (44%)	13 (38%)	0.594
Take a tester	34 (37%)	21 (36%)	13 (38%)	0.800
Keep Narcan/naloxone nearby	32 (34%)	14 (24%)	18 (53%)	0.006
Do you know where you can buy or get Narcan/naloxone?^c^				
Yes	53 (57%)	27 (46%)	26 (76%)	0.013
No	29 (31%)	23 (39%)	6 (18%)	

^a^Categories are not mutually exclusive

^b^Restricted to persons who reported heroin use in the past 6 months (*n* = 43)

^c^Among the participants who answered “yes” to “Have you ever heard of naloxone, a medication also known as Narcan?” (*n* = 82)

After the brief training, overall willingness to use rapid fentanyl test strips was high among participants who had ever overdosed and those who had not; overall, 95% agreed or strongly agreed that they planned to use the provided rapid fentanyl take-home test strips. Almost all study participants (*n* = 92, 99%) reported that it would be easy to use the fentanyl testing strips. Among the participants who were trained to use rapid fentanyl test strips on their urine (*n* = 40), over half (56%) reported that they would prefer to use a test that could detect fentanyl in drugs dissolved in water before using them. Nearly three quarters of the overall sample (*n* = 66, 71%) endorsed “yes” when asked “Do you think your friends would be interested in using the fentanyl testing strips?”.

## Discussion

Contrary to our primary hypothesis, nearly all participants reported high willingness to use the fentanyl test strips, regardless of previous overdose history. The majority of the study participants reported wanting to know if their drug supply was contaminated with fentanyl. All study participants reported feeling confident in their ability to test their drugs or urine for fentanyl after a brief skill-based training. Nearly all participants reported wanting to know if there was fentanyl in their drugs before taking them, indicating a clear preference for testing their drugs or residue directly rather than testing their urine after drug use. These findings suggest that rapid drug testing is an acceptable, low threshold intervention that could be used to address concerns associated with emerging adulterants (e.g., fentanyl) in the illicit opioid drug supply.

Despite the fact that reporting a history of overdose was not associated with higher willingness to use the fentanyl testing strips, prior non-fatal overdose is among the strongest predictors of future overdose death [31, 32]. In this study, young adults who had ever experienced an overdose were more likely to have been homeless, have an arrest history, and report more frequent and injection drug use, among other risk factors. If fentanyl rapid test strips are found to be an effective harm reduction intervention, making the technology readily available to individuals who demonstrate these characteristics may be a high public health priority. Although off-label use of fentanyl testing strips has not been approved by the US Food and Drug Administration (FDA), they are already being used at syringe exchange programs in areas with a high burden of fentanyl-related overdoses, such as Vancouver, New York City, and Boston [33, 34]. Rapid fentanyl drug testing may also be incorporated into drug checking services provided at nightclubs and music venues seen in Europe [35].

While there has been some concern raised about the ability of the rapid fentanyl test strips to detect novel fentanyl analogs [34], the tests have been shown to detect the most common fentanyl analogs currently circulating in the illicit drug supply, such as carfentanil and acetyl fentanyl [29]. Further studies should examine which fentanyl analogs are able to be detected in urine or drugs and to what degree of sensitivity. Nonetheless, in light of concerns regarding false negatives, rapid fentanyl test strips should be distributed alongside information about what to do regardless of whether the drug tests positive for fentanyl, such as using with someone nearby who is capable of calling emergency medical services. In general, our results suggest that rapid drug tests might be an acceptable intervention for young adults who use drugs for identifying adulterants in the drug illicit supply.

Future research is needed to determine if using fentanyl rapid testing strips will lead to desired behavioral changes outlined in the IMB model, such as obtaining naloxone and using drugs with others who can call an ambulance if an overdose occurs [36, 37]. A future manuscript will discuss follow-up data and whether knowledge of a drug being contaminated with fentanyl will encourage overdose risk reduction practices. Future research is needed to determine if a higher level of concern about overdose will predict actual rapid test strip utilization or more consistent use patterns. Research is also needed to better understand the feasibility of using the rapid test strips among individuals who do not see themselves as at risk for fentanyl overdose, such as people who buy pills on the street or people who use cocaine. Additional studies are also needed to determine the sensitivity and specificity of using immunoassay tests on drugs directly and in real-world, non-clinical settings.

We are aware of a number of limitations to this study. This is a small pilot study which recruited a convenience sample from a region highly impacted by the fentanyl overdose epidemic; therefore, this study may not be generalizable to other settings. Additionally, in some regions, fentanyl contamination is widespread throughout the drug supply, and it has yet to be determined how that may affect uptake of rapid fentanyl test strips. Third, we only assessed lifetime overdose history generally and did not ask participants about their experiences with opioid overdose specifically. It is possible that participants who have previously experienced an opioid overdose, or had an overdose they thought was caused by fentanyl, may be more willing to use the fentanyl testing strips. Finally, this study relied on self-report, which may be subject to socially desirable reporting. However, evidence has shown a high association between willingness to use a harm reduction program and subsequent uptake of the intervention among people who use drugs [38].

## Conclusions

In summary, this study assessed the feasibility and acceptability of take-home rapid fentanyl tests. We found a high willingness to use take-home rapid fentanyl test strips among young people who use drugs and are at risk for accidental fentanyl overdose. Initial results suggest that rapid fentanyl test strips may be an acceptable harm reduction intervention for communities facing growing rates of fentanyl overdoses. Study follow-up is ongoing to determine whether, how, and under what circumstances participants used these rapid test strips and if positive test results contribute to positive changes in overdose risk behavior.

## References

[1] Rudd RA, Seth P, David F, Scholl L (2016). Increases in drug and opioid-involved overdose deaths—United States, 2010–2015. MMWR Morb Mortal Wkly Rep.

[2] Centers for Disease Control and Prevention, National Center for Health Statistics, National Vital Statistics System. Provisional counts of drug overdose deaths, as of 8/6/2017 [Internet]. 2017. Available from: https://www.cdc.gov/nchs/data/health_policy/monthly-drug-overdose-death-estimates.pdf. Accessed 6 Feb 2018.

[3] Carlson RG, Nahhas RW, Martins SS, Daniulaityte R (2016). Predictors of transition to heroin use among initially non-opioid dependent illicit pharmaceutical opioid users: a natural history study. Drug Alcohol Depend.

[4] Martins SS, Sarvet A, Santaella-Tenorio J, Saha T, Grant BF, Hasin DS (2017). Changes in US lifetime heroin use and heroin use disorder: prevalence from the 2001–2002 to 2012–2013 National Epidemiologic Survey on alcohol and related conditions. JAMA Psychiatry.

[5] Compton WM, Jones CM, Baldwin GT (2016). Relationship between nonmedical prescription-opioid use and heroin use. N Engl J Med.

[6] Cicero TJ, Ellis MS, Surratt HL (2012). Effect of abuse-deterrent formulation of OxyContin. N Engl J Med.

[7] Dart RC, Surratt HL, Cicero TJ, Parrino MW, Severtson SG, Bucher-Bartelson B (2015). Trends in opioid analgesic abuse and mortality in the United States. N Engl J Med.

[8] O’Donnell JK, Gladden RM, Seth P (2017). Trends in deaths involving heroin and synthetic opioids excluding methadone, and law enforcement drug product reports, by census region—United States, 2006–2015. MMWR Morb Mortal Wkly Rep.

[9] Somerville NJ. Characteristics of fentanyl overdose—Massachusetts, 2014–2016. MMWR Morb Mortal Wkly Rep [Internet]. 2017;66 Available from: 10.15585/mmwr.mm6614a2. Accessed 6 Feb 2018. 10.15585/mmwr.mm6614a2 PMC5657806 28406883

[10] Drug Enforcement Administration. National drug threat assessment summary. Washington, DC: US Department of Justice, Drug Enforcement Administration; 2016. Nov. Available from: https://www.hsdl.org/?abstract&did=797265. Accessed 6 Feb 2018.

[11] Counterfeit Prescription Pills Containing Fentanyls. A global threat. DEA intelligence brief. Washington, DC: US Department of Justice, Drug Enforcement Administration; 2016. Available from: https://www.hsdl.org/?abstract&did=796541. Accessed 6 Feb 2018.

[12] Influx of Fentanyl-Laced Counterfeit Pills and Toxic Fentanyl-Related Compounds Further Increases Risk of Fentanyl-Related Overdose and Fatalities. HAN health advisory; 2016. Centers for Disease Control and Prevention; 2016. Available from: https://emergency.cdc.gov/han/han00395.asp. Accessed 6 Feb 2018.

[13] Mack KA, Jones CM, Ballesteros MF (2017). Illicit drug use, illicit drug use disorders, and drug overdose deaths in metropolitan and nonmetropolitan areas—United States. MMWR Surveill Summ.

[14] Dowell D, Noonan RK, Houry D. Underlying factors in drug overdose deaths. JAMA [Internet]. 2017; Available from: 10.1001/jama.2017.15971. Accessed 6 Feb 2018. 10.1001/jama.2017.15971 PMC6007807 29049472

[15] Gladden RM, Martinez P, Fentanyl Law SP (2016). Enforcement submissions and increases in synthetic opioid-involved overdose deaths—27 states, 2013–2014. MMWR Morb Mortal Wkly Rep.

[16] Office of the Chief Coroners. Fentanyl-Detected Illicit Drug Overdose Deaths (January 1, 2012 to August 31, 2017) [Internet]. British Columbia Coroners Service; 2017 Oct. Available from: https://www2.gov.bc.ca/assets/gov/public-safety-and-emergency-services/death-investigation/statistical/fentanyl-detected-overdose.pdf. Accessed 6 Feb 2018.

[17] Rudd RA, Aleshire N, Zibbell JE, Matthew Gladden R (2016). Increases in drug and opioid overdose deaths—United States, 2000–2014. Am J Transplant.

[18] Jones CM, Logan J, Gladden RM, Bohm MK (2015). Vital signs: demographic and substance use trends among heroin users—United States, 2002–2013. MMWR Morb Mortal Wkly Rep.

[19] Marshall BDL, Krieger MS, Yedinak JL, Ogera P, Banerjee P, Alexander-Scott NE (2017). Epidemiology of fentanyl-involved drug overdose deaths: a geospatial retrospective study in Rhode Island, USA. Int. J. Drug Policy..

[20] Frank D, Mateu-Gelabert P, Guarino H, Bennett A, Wendel T, Jessell L (2015). High risk and little knowledge: overdose experiences and knowledge among young adult nonmedical prescription opioid users. Int. J. Drug Policy..

[21] Marshall BDL, Green TC, Yedinak JL, Hadland SE (2016). Harm reduction for young people who use prescription opioids extra-medically: obstacles and opportunities. Int J Drug Policy.

[22] Ciccarone D, Ondocsin J, Mars SG (2017). Heroin uncertainties: exploring users’ perceptions of fentanyl-adulterated and -substituted “heroin”. International Journal of Drug Policy.

[23] Akins T. Drug checking at Insite shows potential for preventing fentanyl-related overdoses [Internet]. Vancouver Coastal Health. 2017 [cited 2017 Nov 9]. Available from: http://www.vch.ca/about-us/news/news-releases/drug-checking-at-insite-shows-potential-for-preventing-fentanyl-related-overdoses. Accessed 6 Feb 2018.

[24] Mark Lysyshyn, Carolyn Dohoo, Sara Forsting, Thomas Kerr, Ryan McNeil. Evaluation of a fentanyl drug checking program for clients of a supervised injection site, Vancouver, Canada. [cited 2017 Nov 9]. Available from: https://www.hri.global/files/2017/05/14/188_Lysyshyn.docx. Accessed 06 Feb 2018. 10.1186/s12954-018-0252-8 PMC6131768 30200991

[25] Anderson ES, Wagstaff DA, Heckman TG, Winett RA, Roffman RA, Solomon LJ (2006). Information-motivation-behavioral skills (IMB) model: testing direct and mediated treatment effects on condom use among women in low-income housing. Ann Behav Med.

[26] Brown W, Carballo-Diéguez A, John RM, Schnall R (2016). Information, motivation, and behavioral skills of high-risk young adults to use the HIV self-test. AIDS Behav.

[27] Macmadu A, Carroll JJ, Hadland SE, Green TC, BDL M (2017). Prevalence and correlates of fentanyl-contaminated heroin exposure among young adults who use prescription opioids non-medically. Addict Behav.

[28] Amlani A, McKee G, Khamis N, Raghukumar G, Tsang E, Buxton JA (2015). Why the FUSS (Fentanyl urine screen study)? A cross-sectional survey to characterize an emerging threat to people who use drugs in British Columbia, Canada. Harm Reduct J.

[29] BTNX | Fentanyl (FYL) Test Strip [Internet]. [cited 2017 Oct 18]. Available from: http://www.btnx.com/Product?id=2002. Accessed 6 Feb 2018.

[30] Armenian P, Vo KT, Barr-Walker J, Lynch KL. Fentanyl, fentanyl analogs and novel synthetic opioids: a comprehensive review. Neuropharmacology [Internet]. 2017; Available from: 10.1016/j.neuropharm.2017.10.016. Accessed 6 Feb 2018. 10.1016/j.neuropharm.2017.10.016 29042317

[31] Caudarella A, Dong H, Milloy MJ, Kerr T, Wood E, Hayashi K (2016). Non-fatal overdose as a risk factor for subsequent fatal overdose among people who inject drugs. Drug Alcohol Depend.

[32] Coffin PO, Tracy M, Bucciarelli A, Ompad D, Vlahov D, Galea S. Identifying injection drug users at risk of nonfatal overdose. Acad Emerg Med. 2007;14:616–23. 10.1197/j.aem.2007.04.005 17554010

[33] Stewart B. Demand for fentanyl test strips booms—but test is not widely available [Internet]. CBC News. 2017 [cited 2017 Nov 29]. Available from: http://a.msn.com/01/en-ca/BBFRkAq?ocid=se. Accessed 6 Feb 2018.

[34] Bebinger M. As Fentanyl Deaths Rise, An Off-Label Tool Becomes A Test For The Killer Opioid [Internet]. WBUR. 2017 [cited 2017 Oct 17]. Available from: http://www.wbur.org/commonhealth/2017/05/11/fentanyl-test-strips. Accessed 6 Feb 2018.

[35] Brunt TM, Nagy C, Bücheli A, Martins D, Ugarte M, Beduwe C (2017). Drug testing in Europe: monitoring results of the Trans European Drug Information (TEDI) project. Drug Test Anal.

[36] McKee G, Amlani A, Buxton - BCMJ J, 2015. Illicit fentanyl: An emerging threat to people who use drugs in BC. researchgate.net [Internet]. 2015; Available from: http://www.bcmj.org/bc-centre-disease-control/illicit-fentanyl-emerging-threat-people-who-use-drugs-bc. Accessed 6 Feb 2018.

[37] Hawk KF, Vaca FE, D’Onofrio G (2015). Reducing fatal opioid overdose: prevention, treatment and harm reduction strategies. Yale J Biol Med.

[38] DeBeck K, Kerr T, Lai C, Buxton J, Montaner J, Wood E (2012). The validity of reporting willingness to use a supervised injecting facility on subsequent program use among people who use injection drugs. Am J Drug Alcohol Abuse.

